# The Importance of a Multidisciplinary Approach in the Management of a Patient with Type I Gaucher Disease

**DOI:** 10.3390/diseases6030069

**Published:** 2018-07-26

**Authors:** Miguel-Ángel Torralba-Cabeza, Susana Olivera-González, José-Luis Sierra-Monzón

**Affiliations:** Aragon Health Research Institute (ISS Aragón), Department of Internal Medicine, Unit of Rare Disorders, “Lozano Blesa” University Hospital, 15th San Juan Bosco Avenue, 50009 Zaragoza, Spain; susana.olivera@yahoo.es (S.O.-G.); jlsierram@hotmail.com (J.-L.S.-M.)

**Keywords:** multidisciplinary, Gaucher, genotype/phenotype correlation, genetic counselling

## Abstract

Managing the multisystemic symptoms of type I Gaucher Disease (GD) requires a multidisciplinary team approach that includes disease-specific treatments, as well as supportive care. This involves a range of medical specialists, general practitioners, supportive care providers, and patients. Phenotype classification and the setting of treatment goals are important for optimizing the management of type I GD, and for providing personalized care. The ability to classify disease severity using validated measurement tools allows the standardization of patient monitoring, and the measurement of disease progression and treatment response. Defining treatment goals is useful to provide a benchmark for assessing treatment response and managing the expectations of patients and their families. Although treatment goals will vary depending on disease severity, they include the stabilization, improvement or reversal (if possible) of clinical manifestations. Enzyme replacement therapy (ERT) is the standard care for patients with type I GD, but a novel substrate reduction therapy (SRT), Eliglustat, has demonstrated safety and efficacy in selected patients. To ensure that treatment goals are being achieved, regular and comprehensive follow up are necessary.

## 1. Introduction

Gaucher disease (GD) is a rare autosomal recessive condition within the group of lysosomal diseases caused by mutations in the GBA gene encoding the enzyme acid B-glucosidase or B-glucocerebrosidase (EC 3.2.1.45) [1]. A complete or partial deficiency of this protein results in the accumulation of glucosylceramide (GC) into the macrophages of the reticuloendothelial system. GC represents the last link in the chain degradation of complex lipids, which are part of the membranes of senescent hematologic cells. GD has been classically divided in three main subtypes from a phenotypic point of view: nonneuronopathic type I (230800), acute neuronopathic type II (230900), and subacute neuronopathic type III (231000). Type II is less common and is characterized by rapidly progressive neurovisceral storage disorders and early death. Type III is less rapidly progressive but also involves neurovisceral problems and premature death. Type I is the most common form of GD characterized by marked variability in phenotypic expression and lack of neurologic involvement (except some cases with Parkinsonism) [2]. All three forms of GD are caused by mutations in the GBA gene. At present, more than 300 mutations have been described, which are included in the databases [3]. Of these, more than 80% are substitutions of a single nucleotide and the rest correspond to complex alleles. The four mutant alleles N370S (c.1226 A > G), L444P (c.1448 T > C), 84GG (c.84dupG) and ISV2 + 1 (c.115 + 1G > A) are the most prevalent worldwide. GC accumulation affects the liver, spleen, and bone marrow, but especially important is bone involvement since 85% of the patients present diffuse osteoporosis, or medullary expansion of the femur (Erlenmeyer flask deformity), or localized osteolysis or avascular necrosis (including sclerosis and osteitis) in long bones, or diffuse destruction with epiphyseal collapse in hips, shoulders, vertebrae or sacroiliac [2]. Because type I GD accounts for 95% of cases worldwide it is especially important to remember that as we have progressed in the knowledge of the pathophysiology and treatment, the expectation and quality of life have been increasing. There have also been important technological advances and medical research involved in the diagnosis and monitoring of patients. The continuous development makes it practically impossible to gather in a single professional all the knowledge and skills required for its approach. This scenario forces health professionals to work in a different way, in which the opinions of the different medical specialties are combined and articulated to treat and understand the patient with type I GD and his family. Several meetings of experts have been carried out for years to address this new vision of type I GD, but there is no publication to date which presented in an orderly manner how to carry out a multidisciplinary approach. After diagnosing a patient with type I GD, clinical management has two aims: (1) How to optimize the management and follow-up of the patient when there is a variety of specialists involved in patient care, and (2) in particular, how to achieve a balance between standardized or individualized treatment. In this paper, we will try to answer if a multidisciplinary approach improves the management of patients with type I GD, and to what extent. From a historical point of view, the first teams that began patient care using a multidisciplinary approach were Oncologists, and it is now a widely held view that the treatment of most cancers has benefitted from this integrated approach. In addition, patient satisfaction and efficiency are improved [4,5]. If this is true for cancer, should we copy this model for type I GD? To elaborate this article, we performed a PubMed advanced search using the keywords “multidisciplinary” and “type I Gaucher Disease“, without finding any reference. Then, we selected the most important articles written from 1990 to today.

## 2. Optimizing Management and Follow-Up of Patients with Type I Gaucher Disease

**Figure 1:**
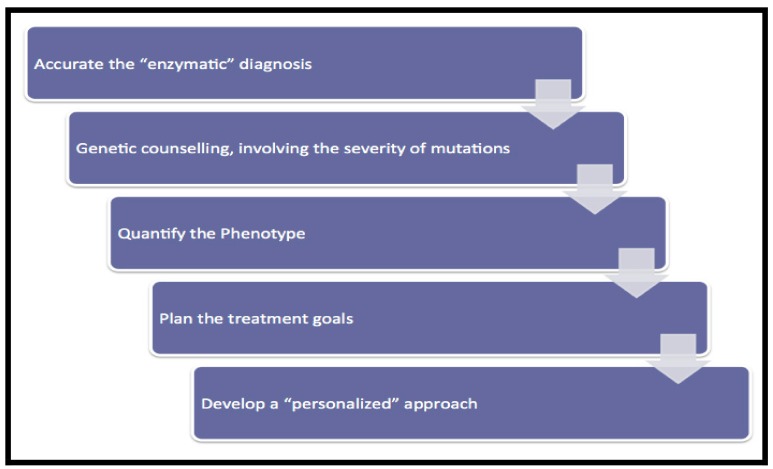
Management program of type I GD patients.

In GD there are a number of critical points in the management of patients which have been described by previous authors [6] (Figure 1).

### 2.1. Enzymatic Analysis

In GD, this represents the only method for a confirmed diagnosis. Detection of a low enzymatic Activity of acid β-glucosidase in peripheral blood leukocytes compared with healthy controls is the “gold standard” for diagnosis, and not bone marrow aspiration [7,8]. The downside of this technique is that it does not allow the identification of healthy carriers.

### 2.2. Genetic Counselling

Due to the characteristics of the GBA gene and its pseudogene, as well as to the most frequently used diagnostic techniques prior to the standardization of mass sequencing, it is especially important to carry out a complete family study. This is especially important in prevalent mutations such as c.1226A > G (N370S) and c.1263del55 (55 bp deletion in exon 9) [9].

GD is an autosomal recessive condition, but challenges in providing Genetic Counselling for an autosomal recessive inheritance are: (1) Lack of family history; (2) Occasionally, autosomal recessive conditions occur in sequential generations; (3) If the biologic father of an affected individual is someone other than the person assumed to be the father, misleading carrier test results might occur (the apparent father would usually not be a carrier) and risk of additional affected children could be misstated; (4) Possibility for the phenomenon called “uniparental disomy”; (5) Although rare, de novo mutations can account for ~1% of gene mutations [10].

### 2.3. Severity of Mutations

Mutations that cause a truncated protein or an alteration in protein stability are the most serious. But in the impact of acid B-glucosidase has been studied using protocols of site-directed mutagenesis and expression of mutant alleles in different cells, providing information about residual enzymatic activities [11]. Recently, we published a study in Spanish GD patients involving the phenotype (at diagnosis) and the genotype correlation according to the residual enzymatic activities [12]. We found that, at least in Spanish population, a lower residual activity results in a more severe phenotype and vice versa, and the absence of this correlation in some siblings can be attributed to epigenetic phenomena and the existence of modifier genes [13].

### 2.4. Phenotypic Quantification

Quantification of the phenotype is the most important tool for generalized and personal assessment. In 1992 Zimran et al. published the Severity Score Index (SSI), useful for type I, II, and III GD patients. This score facilitates the classification of GD patients into those with mild (<10 points), moderate (11–25 points) or severe (>25 points) disease, and it has a great utility at the time of diagnosis [14]. In 2008 Di Rocco et al. published the Gaucher Severity Score Index for type I patients (GauSS-I). GauSS-I involves a maximum of 42 points distributed over six domains (skeletal, haematological, biomarkers, visceral, lung, and neurological) with unequally weighted parameters that allows its use each time that patients come to the clinic [15]. Recently, Weinreb et al. published a very simple score in 2010: The Gaucher Disease type I severity scoring system (GD-DS3), which focuses on bone, hematologic, and visceral aspects. This has proven a useful computer-based tool with a maximum of 19 points after the addition of the averages for each domain [16]. These scoring systems are excellent instruments for quantifying the phenotype and response to therapy or change of dosage. Additionally, biomarkers can assist in the diagnosis and long-term monitoring of type 1 GD, often providing an early warning signal of disease complications. Biomarkers are proteins that arise from Gaucher cells, and their plasma levels offer insights into total disease burden. Irrespective of the investigation into new biomarkers, chitotriosidase [17], CCL18/PARC [18], and recently Glucosylsphingosine, are the most useful [19].

### 2.5. Treatment Goals

**Figure 2:**
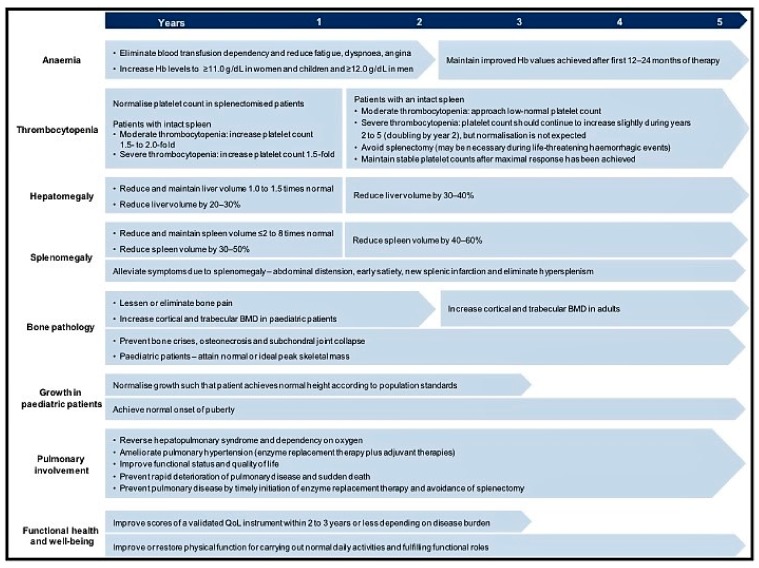
Adaptation of the therapeutic goals to be achieved in type I GD patients, according to Pastores et al.

Generally speaking, the objectives in GD are to: (1) stabilize, (2) improve, and (3) reverse (if possible) clinical signs and symptoms. In 2004, Pastores et al. published therapeutic goals for type I GD patients [20] (Figure 2). These recommendations were recently revised to include the prevention of long-term complications or associated comorbidities [21] (i.e., residual skeletal disease, monoclonal gammopathy of undetermined significance, and certain types of cancer, pulmonary hypertension, Parkinson disease, and metabolic syndrome). These additional goals reinforce the importance of taking a multidisciplinary point of view in Gaucher disease, particularly when bone disease; pregnancy, multiple myeloma and Parkinsonism occur. Bone disease is common, frequently severe and unpredictable in terms of presentation, being independent of visceral and hematologic manifestations and the genotype, and is a criterion for starting enzyme replacement treatment (ERT) [22]. For GD women who are pregnant it is important to remember the tendency to worsening anemia (as well decreased platelets), the potential for bone crisis (especially after delivery), and the potential need for ERT dosage adjustment. Especially important are considerations surrounding anesthesia and delivery [23].

For adult GD patients, regular monitoring allows the multidisciplinary team to assess attainment of therapeutic goals. For patients who are not receiving treatment with ERT this should include physical examination, SF-36 survey, blood test and biochemical markers every 12 months. Visceral, skeletal and pulmonary tests must be performed every 12–24 months. For patients on ERT that have not achieved therapeutic goals, blood tests for biochemical markers are recommended every 3 months, plus every 12 months a physical examination, SF-36 survey, and visceral, skeletal and pulmonary assessments. Finally, for patients on treatment who have achieved therapeutic goals, their assessment should be performed every 2 years except for a physical examination and SF-36 survey that should be performed annually [24]. Similar evaluations and monitoring are recommended for pediatric patients, except for a physical examination that should be conducted 6 months to check for height attainment [25].

### 2.6. Implementing “Personalized Medicine”

In general, a multidisciplinary team must achieve the right balance between standardized and individualized care and follow up. Finally, the group (1) defines the problem, (2) decides on goals, (3) gathers information, (4) seeks opinions, (5) discusses and expands the problem, (6) develops potential solutions, (7) offers opinions, (8) evaluates potential solutions and chooses the best one, and (9) summarizes the plan and agrees on distribution of tasks across team members. The keys to success are to involve all team members, have good communication, to work towards treatment standards, and to give patients information through an individualized written report [26,27].

**Figure 3:**
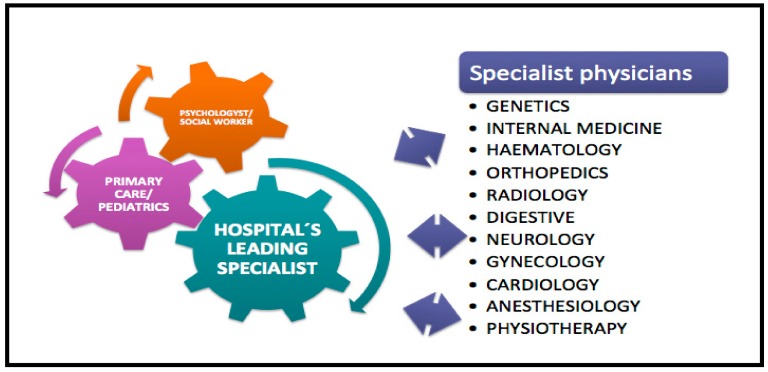
Diagram of a multidisciplinary team in type I GD.

Within a GD multidisciplinary team, it is essential that an expert leads and coordinates all aspects of care including social and psychological support. They need to work closely with the other relevant hospital specialist physicians in order to develop a personal model for treatment and management. A specialist in Internal Medicine is an ideal candidate to lead this approach because of his integrated vision of the patient, but anyone with experience can develop this role which to a certain extent should reflect the organization of each hospital (Figure 3).

**Figure 4:**
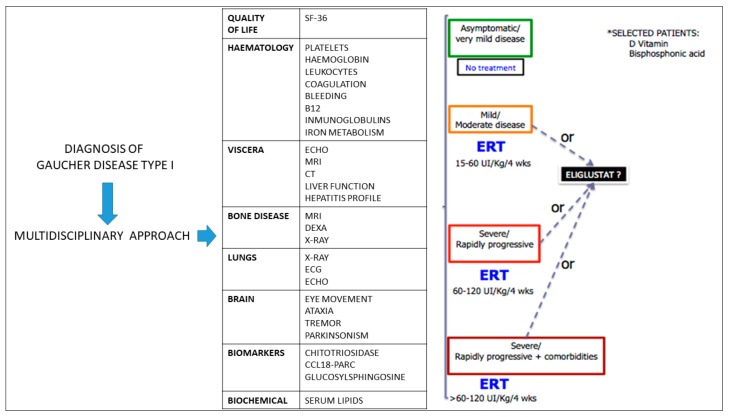
Individualized approach to treatment initiation for type I GD patients.

How can we develop an individualized treatment plan in a patient with newly diagnosed type I GD? ERT with mannose-terminal glucocerebrosidase (imiglucerase and velaglucerase alfa in Europe) is the standard therapeutic approach [28] although some asymptomatic or very mildly affected patients don´t need treatment. Usually, patients are classified as follows [29] (Figure 4):

- Mild/moderate disease: ERT should be started at doses of 15–60 UI/Kg/4 weeks.
- Severe/rapidly progressive: ERT may be required at doses between 60–120 UI/Kg/4 weeks.
- Severe/rapidly progressive in the presence of comorbidities: ERT should be initiated at doses higher than 60–120 UI/Kg/4 weeks.

Substrate reduction therapy (SRT) with miglustat is an alternative for treatment of adults with mild/moderate type I GD for whom ERT is unsuitable [30] but a high percentage of patients discontinue it due to gastrointestinal symptoms or tremor [31]. Recently, a new well-tolerated SRT drug was approved to treat type I GD patients: Eliglustat. With the approval of eliglustat as a first-line therapy, eligible type I GD patients (depending on the CYP2D6 cytochrome genotype) now have the option of a daily oral therapy [28,32]. It will be very important that the multidisciplinary team carefully assess individual patients to determine their appropriateness for eliglustat therapy.

Many patients also require additional drugs or other interventions, such as Vitamin D or bisphosphonates for osteopenia, pain analgesics, orthopaedic surgery and physical therapy for skeletal complications and physical therapy to attenuate portal hypertension, and vasodilator treatment for pulmonary hypertension.

**Figure 5:**
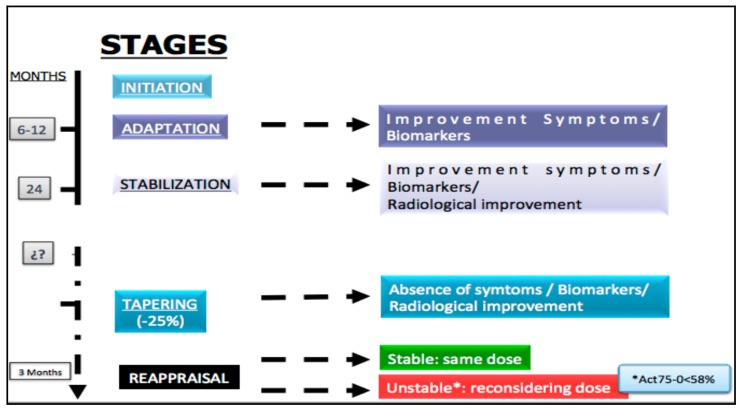
Personalized management of the treatment for type I GD patients.

The personalized management of the treatment for type I GD patients requires five stages [33] (Figure 5):

1. Initiation of selected drug.
2. Adaptation: In this stage a clinical improvement and the biomarkers must be demonstrated. This period of time fluctuates between 6 and 12 months.
3. Stabilization: For most patients it occurs after the first 24 months following the beginning of treatment. Besides the improvement of the clinical aspects and the biomarkers, radiological enhancement must be exhibited.
4. Tapering: After stabilization of the patient, which is defined by the absence of symptoms, normalization of biomarkers and demonstration of a radiological improvement, the tapering stage can be initiated, with a dose reduction of 25% in the case of ERT.
5. Maintenance: 3 Months after modification of the doses, patients must be carefully re-evaluated, according to the “evaluation and monitoring recommendations”. If the patient remains stable the same dosage should be continued, but in an unstable patient the multidisciplinary team should reconsider the appropriate dose. Recently, the usefulness of a new biomarker (Act 75–0 <58%) has been published, which has proved extremely practical for discriminating whether the dose administered is sufficient in the patient with ERT [34].

## 3. Summary

A multidisciplinary care team can only succeed if the following circumstances or support are provided [35]: (1) A whole-team approach, with clarity about the roles and expertise of each team member, including specialists, the general practitioner and allied healthcare professionals including a supportive care provider, who deals with the psychosocial aspects of care; (2) Regular communication among team members; (3) Access to a full range of therapeutic options, irrespective of geographical remoteness, rural or urban healthcare service; (4) Provision of care in line with national standards, and treatment decisions based on adequate information; (5) The patients should be involved in their care discussions and management and should receive timely and appropriate information from the healthcare professionals; and (6) Selecting the right team members.

However, a multidisciplinary team will need to work around common obstacles [35]: (1) Gatekeeping (the mechanism that allows work under some circumstances and blocks them under others); (2) Financial factors; (3) Lack of professional training in a multidisciplinary approach; (4) Logistics (e.g., co-location, available meeting times, and physical resources); (5) Differing reporting requirements for disciplines involved; (6) Lack of formal evaluation criteria; (7) Lack of trust between participating professions; (8) A focus on professional autonomy; and (9) Legislative frameworks which limit the scope of professional practice.

In this rare disorder, a multidisciplinary approach in the management of subjects with GD, especially new patients, is valuable because of delivering benefits on two fronts:

(1) Social: Enhancing access to services, improving the quality of care and lowering overall health care expenditure. Moreover, it allows more efficient use of interventions by setting individualized objectives, varying dosing schedules, and types of therapy.
(2) Medical: This model provides a framework that enriches the clinical interpretation and the applicability of clinical assessment tools which represents the best model for the translation of results from research to clinical practice.
